# Supplementary material for: β-adrenergic receptor signaling evokes the PKA-ASK axis in mature brown adipocytes
Source: PLoS One. 2020 Oct 27;15(10):e0232645. doi: 10.1371/journal.pone.0232645 (PMC7591029; doi:10.1371/journal.pone.0232645)
Supplement: S1 Table — (DOCX) [file pone.0232645.s001.docx]

**Supplementary Table 1 Primer Sequences**

|  | Forward | Reverse | NCBI Ref. Seq. |
| --- | --- | --- | --- |
| Hprt1 | tcctcctcagaccgctttt | cctggttcatcatcgctaatc | NM_013556.2 |
| Pparg | gaaagacaacggacaaatcacc | gggggtgatatgtttgaacttg | NM_001127330.1 |
| Fasn | gctgctgttggaagtcagc | agtgttcgttcctcggagtg | NM_007988.3 |
| Adipoq | ggagagaaaggagatgcaggt | ctttcctgccaggggttc | NM_009605.4 |
| Fabp4 | aagagaaaacgagatggtgacaa | cttgtggaagtcacgccttt | NM_024406.2 |
| Cox8b | ccagccaaaactcccactt | gaaccatgaagccaacgac | NM_007751.3 |
| Cox7a1 | cgaagaggggaggtgactc | agcctgggagacccgtag | NM_009944.3 |
| Ppargc1a | gagcgaaccttaagtgtggaa | tcttggttggctttatgagga | NM_008904.2 |
