# Supplementary figures and images for: β-adrenergic receptor signaling evokes the PKA-ASK axis in mature brown adipocytes
Source: PLoS One. 2020 Oct 27;15(10):e0232645. doi: 10.1371/journal.pone.0232645 (PMC7591029; doi:10.1371/journal.pone.0232645)

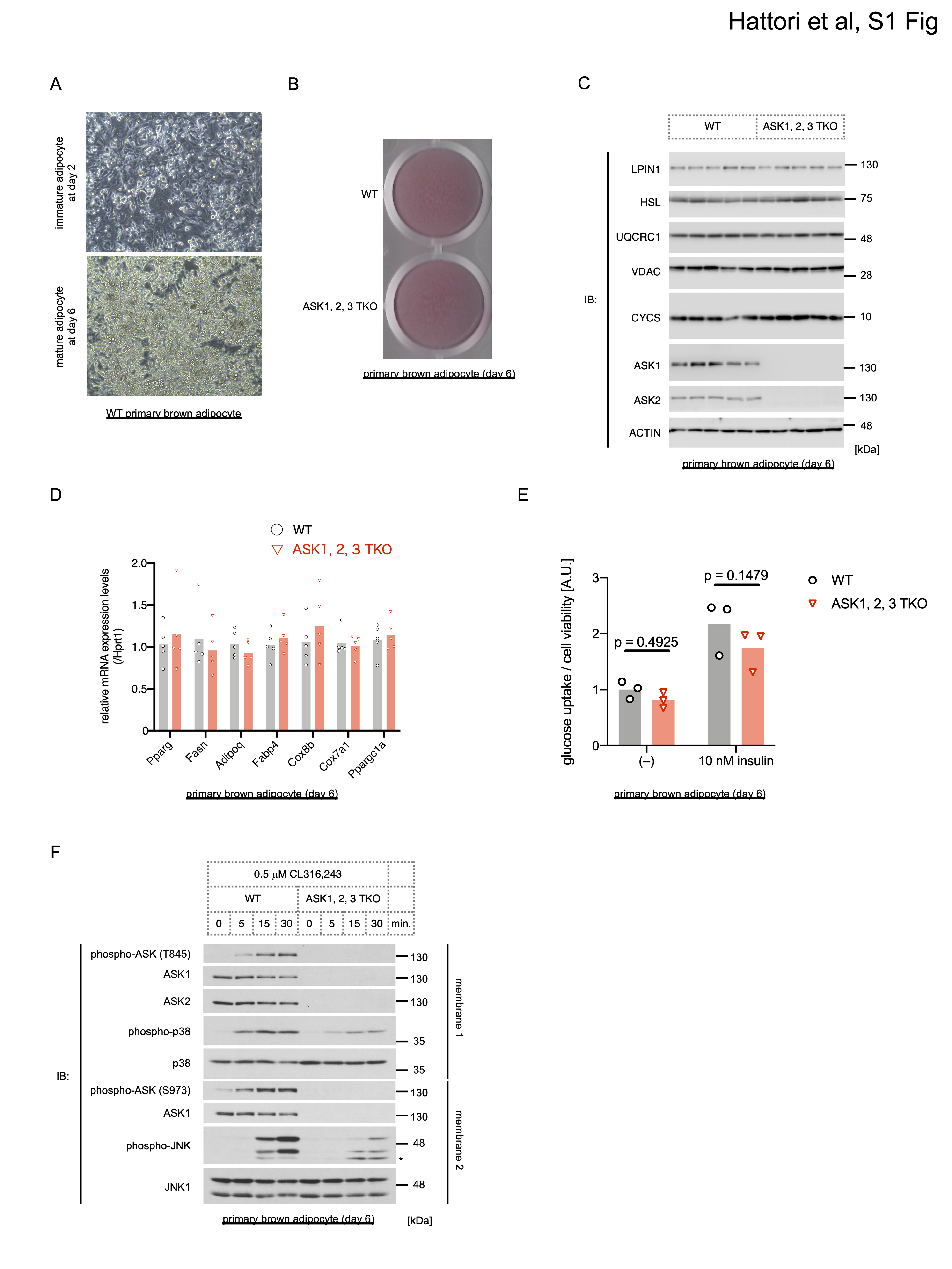

Supplement: S1 Fig — A. Brown adipocytes imaged at day 2 (immature) and day 6 (mature). B-D. Oil red O staining (B), Western blot analysis (C), and qRT-PCR analysis (D) of WT and ASKTKO mature brown adipocytes (day 6). The means and individual data are shown (N = 5) in D. E. 10 nM insulin was treated 30 min before measuring glucose uptake in mature brown adipocytes (day 6). The means and individual data are shown (N = 3). Two-stage linear step-up procedure of Benjamini, Krieger and Yekutieli was used to adjust p-values. F. CL316,243-dependent MAPK activation in mature brown adipocytes (day 6) derived from wild-type and ASKTKO mice, as assessed by Western blotting. (TIF) [file pone.0232645.s002.tif]

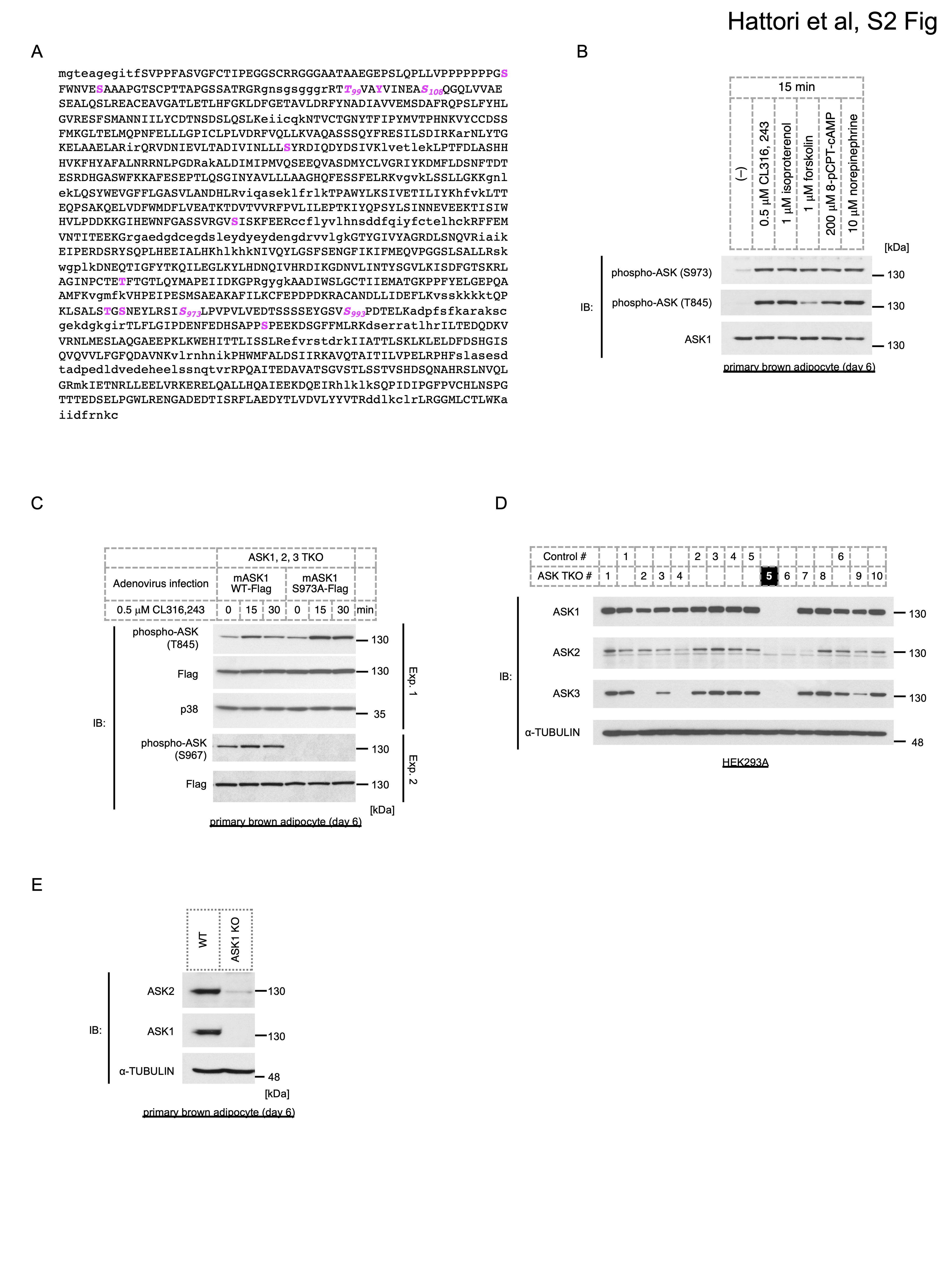

Supplement: S2 Fig — A. The amino acid sequence of mouse ASK1 used in mass spectrometry analysis. Upper case: identified amino acids, lower case: unidentified amino acids, magenta (bold font): identified phosphorylation sites, magenta/bold/italicized (with an amino acid number): sites in which phosphorylation levels are augmented by PKA overexpression. B. Western blot analysis of ASK1 phosphorylation at Ser 973 in response to treatment with a PKA activator for 15 min in mature brown adipocytes (day 6). C. 3’Flag-tagged wild-type ASK1 or mutant ASK1 S973A was overexpressed by adenovirus infection in immature ASKTKO cells (day 2), and differentiated mature brown adipocytes (day 6) were treated with CL316,243 for the indicated time. The phosphorylation of ASK1 was assessed by Western blotting. D. Western blots for ASK1, ASK2, and ASK3 in control and ASKTKO monoclonal HEK293A cells. This is a representative of knockout cell screens. ASKTKO #5 was used in Fig 3C. E. Western blots for ASK2 expression in ASK1KO mature brown adipocytes (day 6). (TIF) [file pone.0232645.s003.tif]

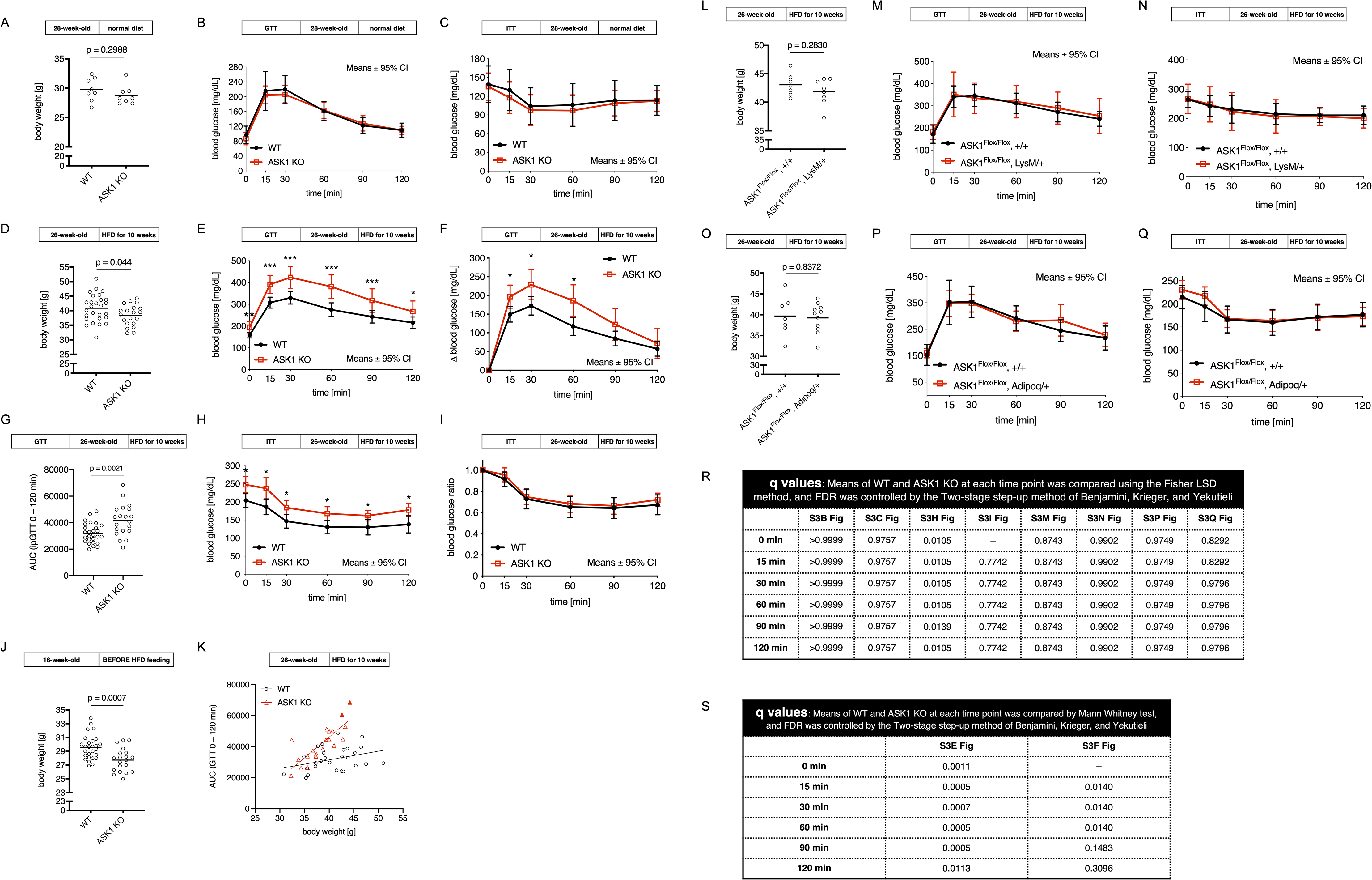

Supplement: S3 Fig — Blood glucose levels (mg/mL) from the glucose tolerance test (GTT) (B, E, F, M, P) and insulin tolerance test (ITT) (C, H, I, N, Q). The body weight of the mice assessed by the GTT and ITT are shown in A, D, L, and O, and unpaired two-tailed Welch’s t-test was used. The body weight of global ASK1KO mice before the start of high-fat diet feeding was plotted and analyzed by unpaired two-tailed Welch's t-test (J). A to C are 28-week-old male WT (N = 8) and global ASK1KO mice (N = 8). D to K are 26-week-old high-fat diet-fed WT (N = 27 for GTT and N = 19 for ITT) and ASK1KO mice (N = 20 for GTT and N = 12 for ITT). L to N are 26-week-old high-fat diet-fed ASK1Flox/Flox; +/+ (N = 7) and ASK1Flox/Flox; LysM-Cre/+ mice (N = 8 for GTT and N = 7 for ITT). O to Q are 26-week-old high-fat diet-fed ASK1Flox/Flox; +/+ (N = 7) and ASK1Flox/Flox; Adipoq-Cre/+ mice (N = 11). The area under the curve (AUC) of S3E Fig was plotted and analyzed by the Mann-Whitney test (G). Correlation of body weight after 10 weeks of high-fat diet exposure and the AUC of the GTT (K). Two data points (filled triangles) represent the data from the mice in which the blood glucose level reached the upper limit of detection. Hence, linear regression curves are shown only for reference. Means ± 95% CI are shown for the GTT and ITT data. The means and individual data are shown for body weight and AUC data. q-values (FDR-adjusted p-values) for the GTT and ITT results were calculated using the Fisher LSD method followed by the two-stage step-up method of Benjamini, Krieger, and Yekutieli (for B, C, H, I, M, N, P, Q). Since the blood glucose level reached the upper limit of detection (600 mg/mL) in 2 mice, the Mann-Whitney test followed by the two-stage step-up method of Benjamini, Krieger, and Yekutieli was used in E and F. See R and S for actual q-values. *q < 0.05, **q < 0.01, ***q < 0.001. (TIF) [file pone.0232645.s004.tif]

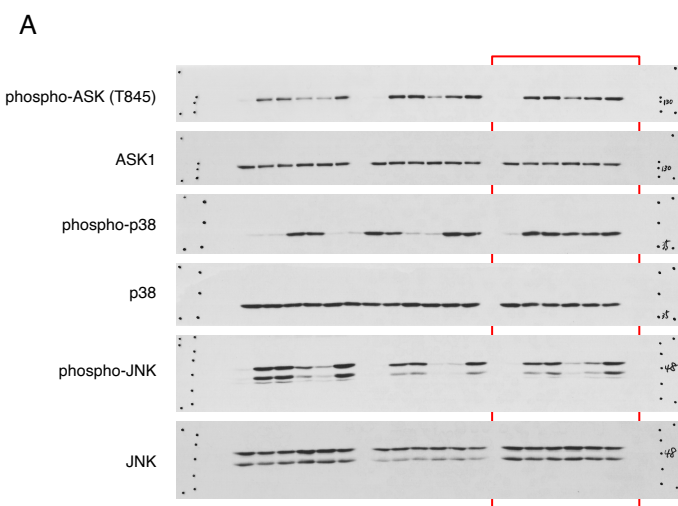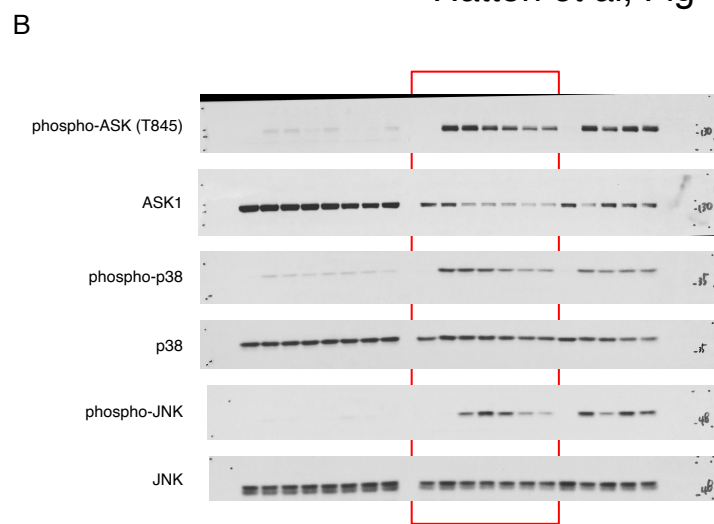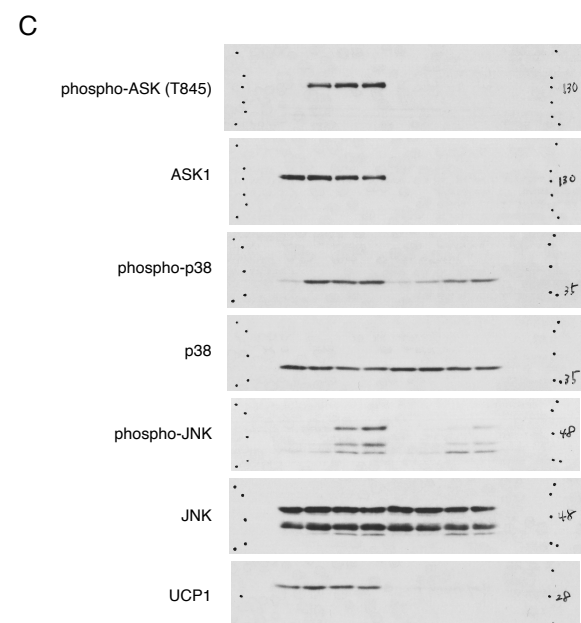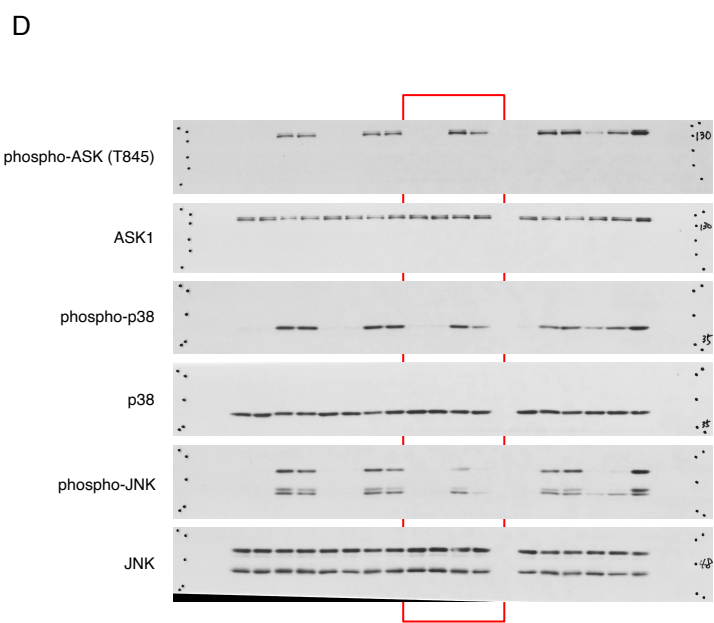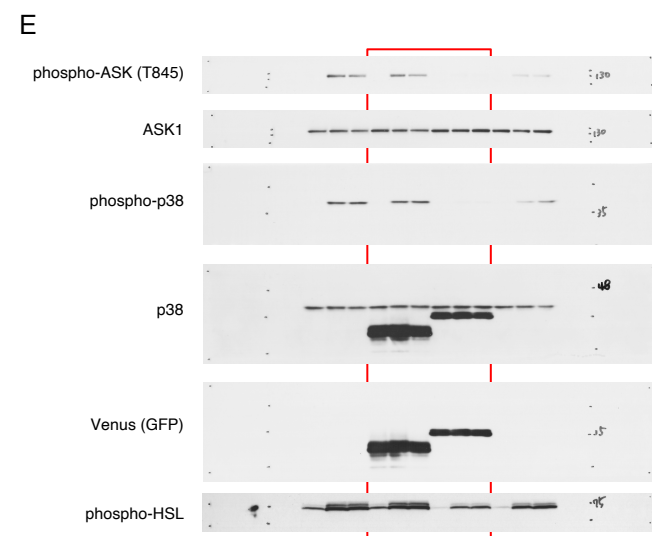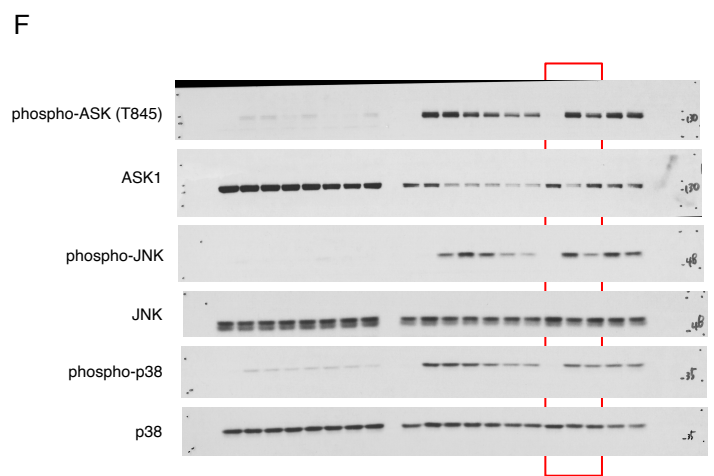

**B**

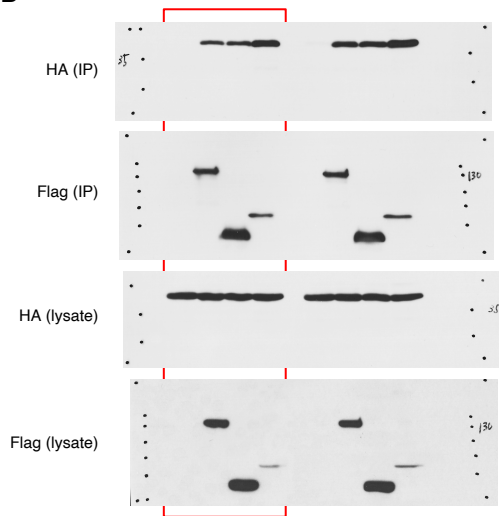

**C**

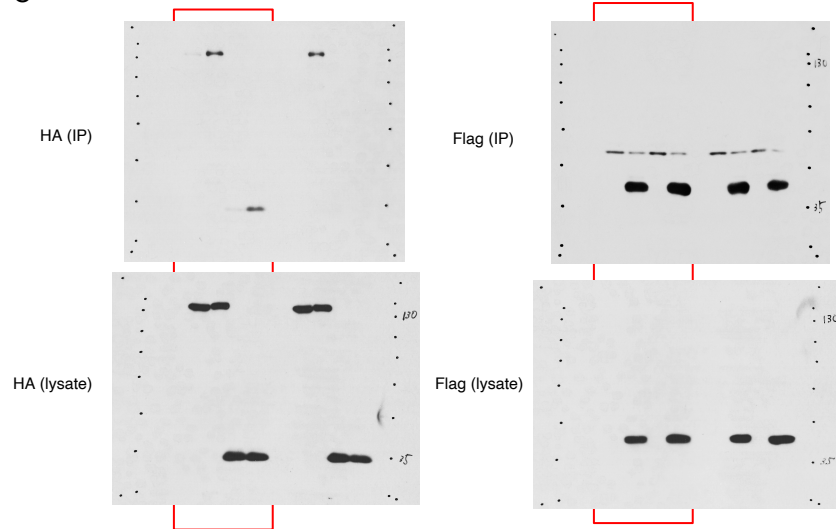

**D**

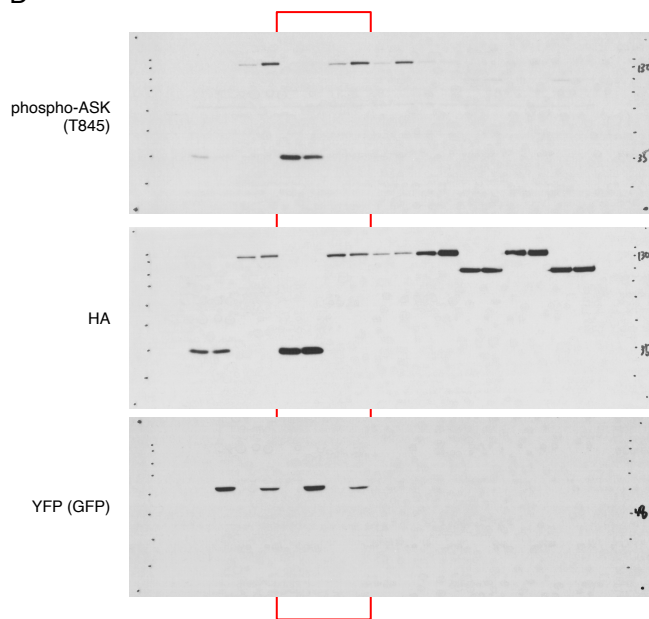

**E**

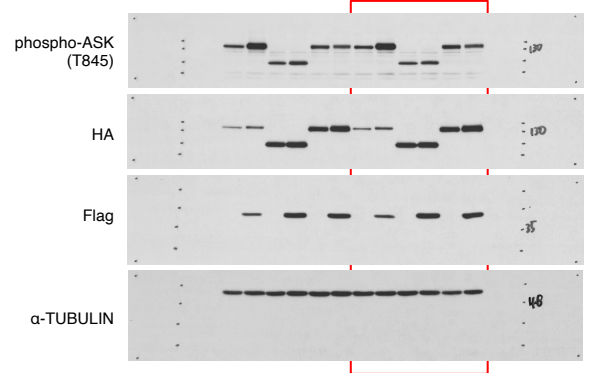

**F**

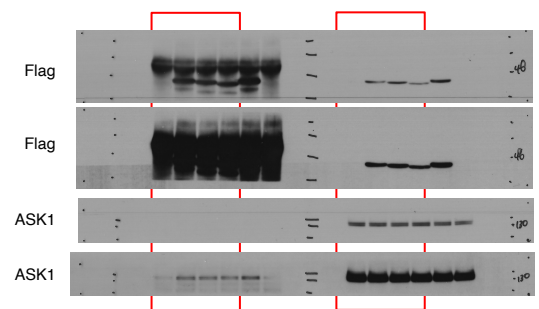

**H**

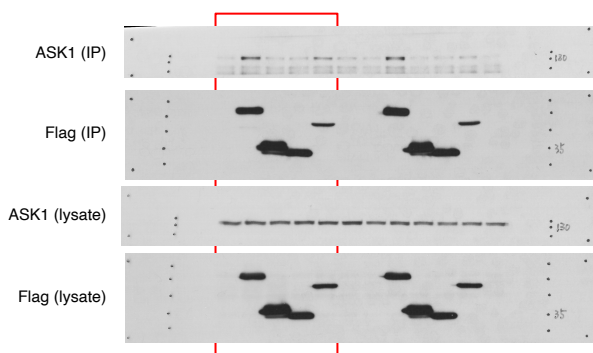

**I**

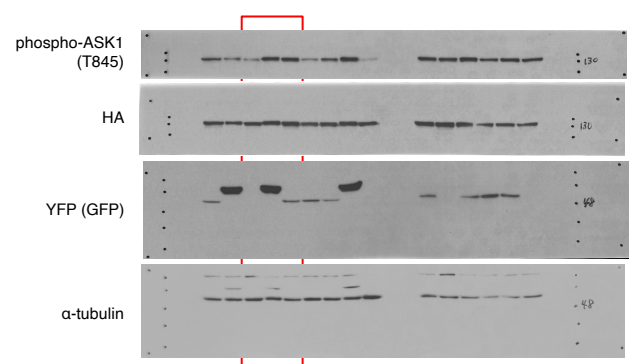

C

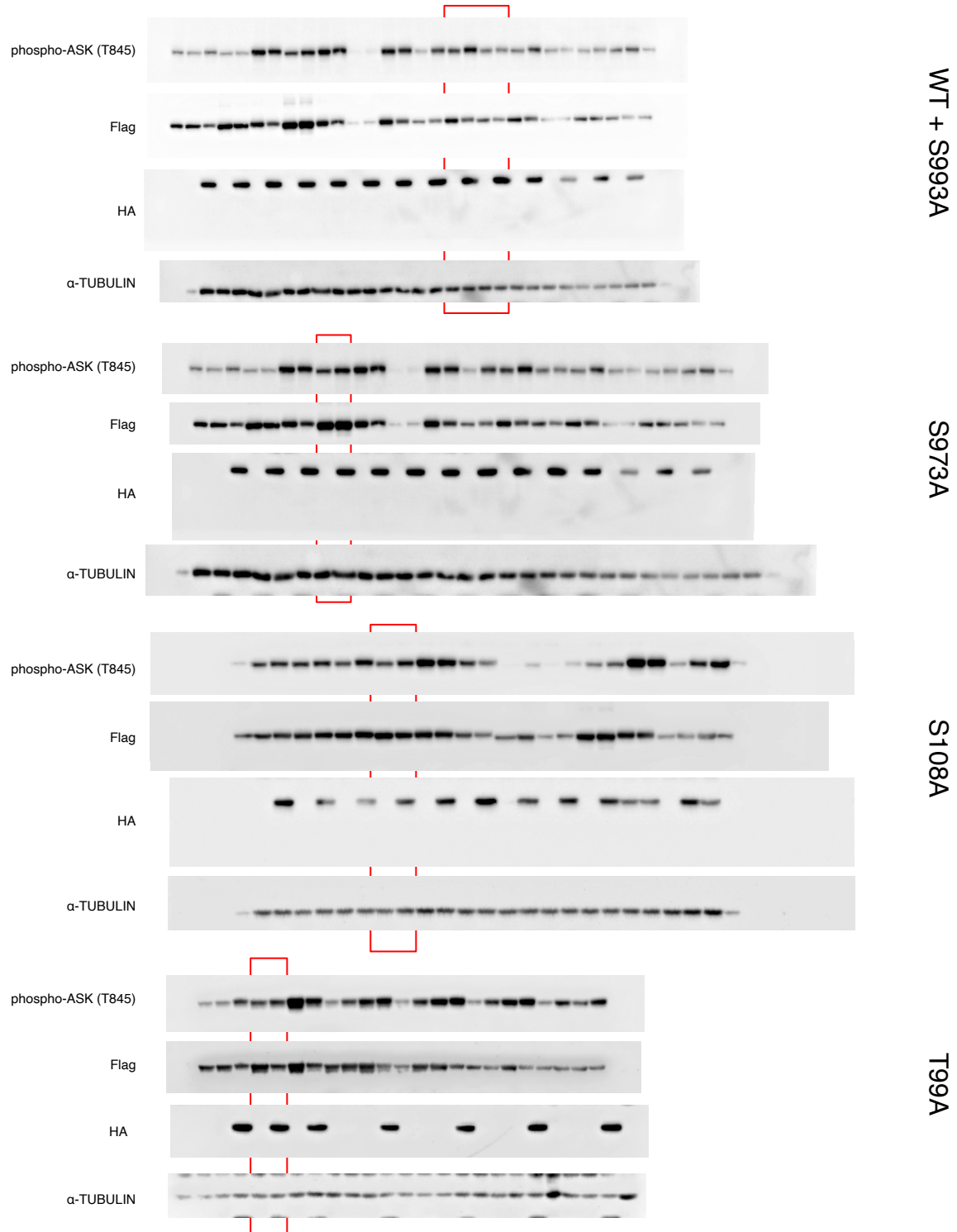

A

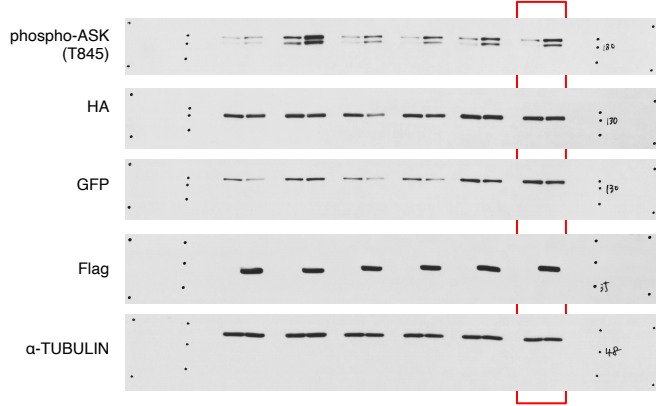

B

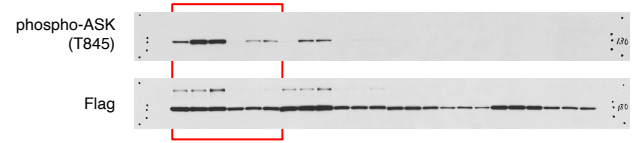

C

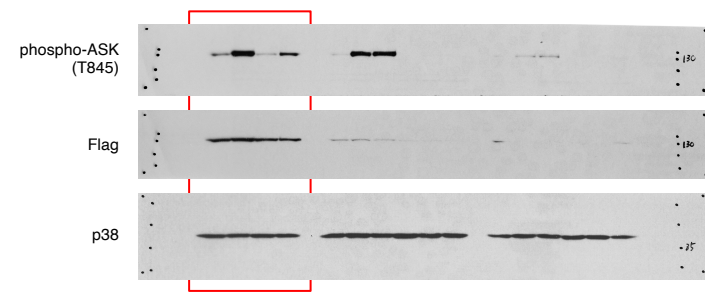

E

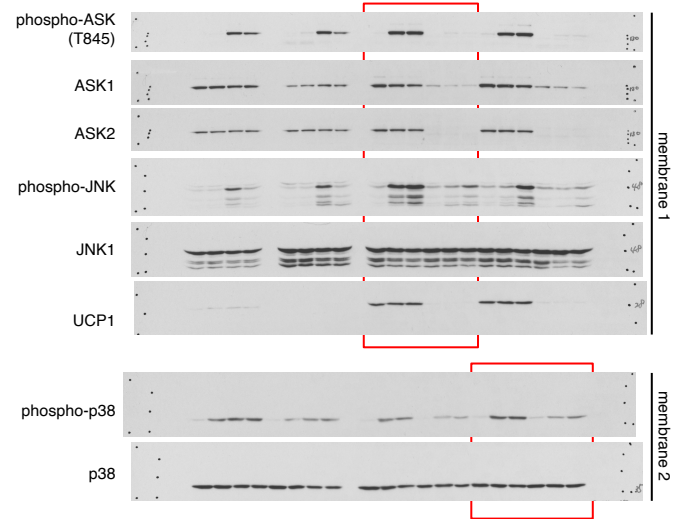

B

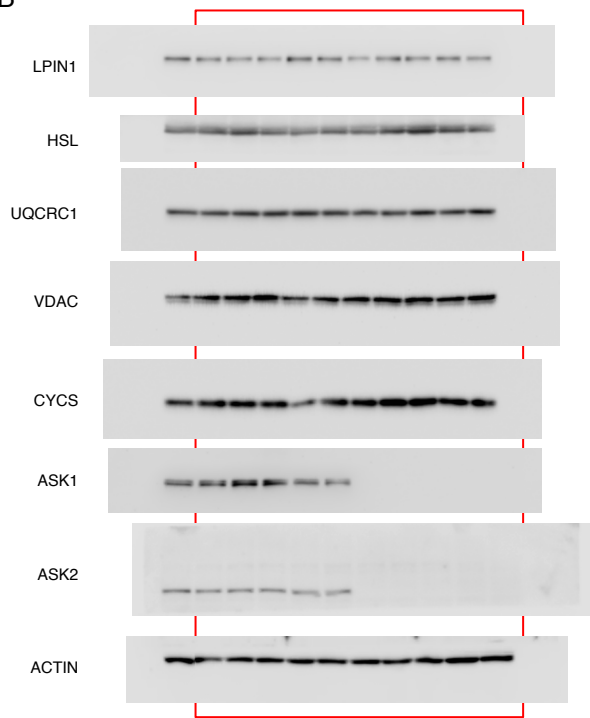

E

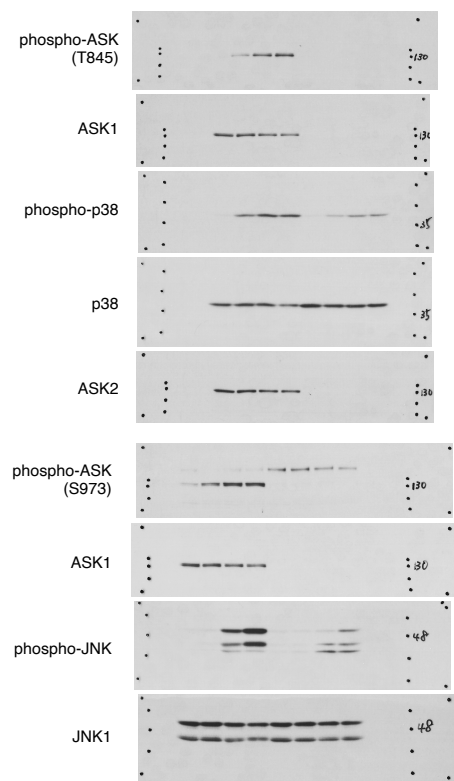

B

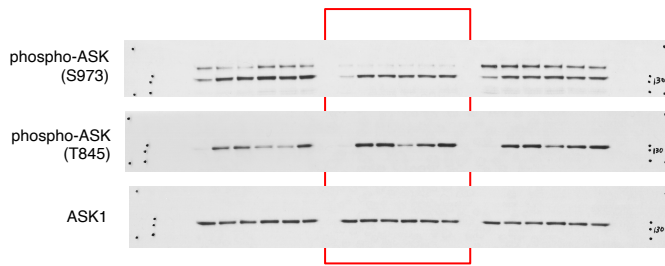

C

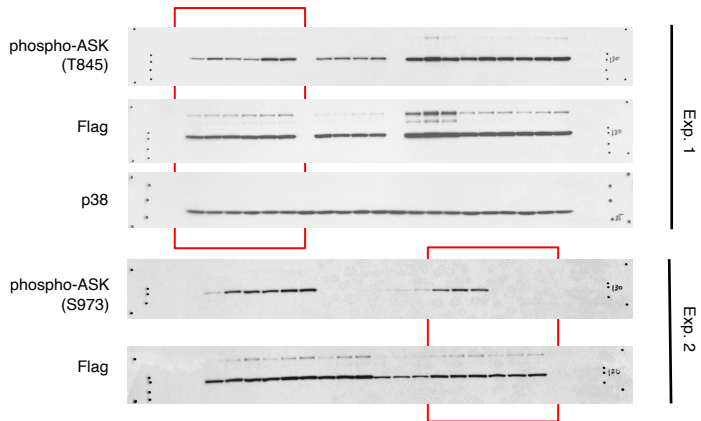

D

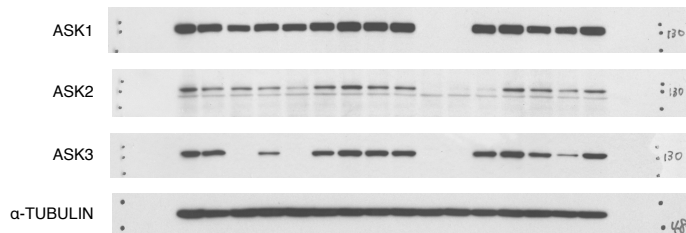

E

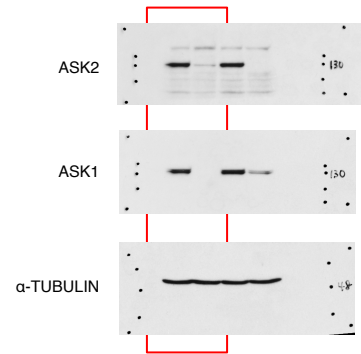

Supplement: S1 Raw images — (PDF) [file pone.0232645.s006.pdf]
